# Imaging lipid domains in cell membranes: the advent of super-resolution fluorescence microscopy

**DOI:** 10.3389/fpls.2013.00503

**Published:** 2013-12-12

**Authors:** Dylan M. Owen, Katharina Gaus

**Affiliations:** ^1^Department of Physics and Randall Division of Cell and Molecular Biophysics, King’s College LondonLondon, UK; ^2^Centre for Vascular Research and Australian Centre for Nanomedicine, University of New South WalesSydney, NSW, Australia

**Keywords:** lipid rafts, membrane microdomains, super-resolution, fluorescence, cell membranes

## Abstract

The lipid bilayer of model membranes, liposomes reconstituted from cell lipids, and plasma membrane vesicles and spheres can separate into two distinct liquid phases to yield lipid domains with liquid-ordered and liquid-disordered properties. These observations are the basis of the lipid raft hypothesis that postulates the existence of cholesterol-enriched ordered-phase lipid domains in cell membranes that could regulate protein mobility, localization and interaction. Here we review the evidence that nano-scaled lipid complexes and meso-scaled lipid domains exist in cell membranes and how new fluorescence microscopy techniques that overcome the diffraction limit provide new insights into lipid organization in cell membranes.

## INTRODUCTION

In the fluid mosaic model (Singer and Nicolson, 1972), the lipid bilayer was originally viewed as a simple 2D fluid in which embedded membrane proteins are able to diffuse freely in the lateral dimension. Many observations however, showed that lipids and membrane proteins are not homogeneously distributed in the plasma membrane. As early as 1987 for example, it was shown that in MDCK cells, sphingolipids first accumulate in the Golgi and are then transported to the apical surface where they are unable to diffuse past tight junctions at cell-cell contact sites (van Meer et al., 1987). If the lipid distribution of the plasma membrane is indeed regulated and non-random, this suggests that biophysical processes exist in cells that cause a lateral organization within the membrane and/or active mechanisms have evolved by which cells sort protein and lipids. It is highly likely that such lateral organization is exploited for specific cell functions.

In 1997, Simons and Ikonen proposed the lipid raft hypothesis in which the phase behavior of different lipid species is exploited to create lateral heterogeneity in the plasma membrane (Parton and Simons, 1995; Simons and Ikonen, 1997). According to this hypothesis, the liquid-disordered phase, formed mainly from unsaturated phospholipids, would coexist in the plasma membrane with a liquid-ordered phases formed from saturated phospholipids and sphingolipids in the presence of cholesterol, which exists in the plasma membrane at concentrations of roughly 30 mole percent. In the ordered phase, a higher degree of conformational order is imposed on the acyl tails of lipids by the rigid ring structure of cholesterol. This results in an increase in the thickness of the lipid bilayer and tighter lipid packing although unlike the gel phase (consisting of saturated lipids in the absence of cholesterol), liquid-ordered bilayer lipids remain laterally mobile. In this model therefore, the plasma membrane is viewed as a “sea” of disordered phase lipids containing stable, ordered phase “islands” or “rafts” enriched in saturated lipids, sphingolipids and cholesterol.

It was then hypothesized that specific membrane proteins would have a high affinity for one phase, thereby partitioning into this phase and being laterally sorted. This would allow lipid rafts to serve as signaling platforms, concentrating some proteins to facilitate their interaction while excluding others (Levental et al., 2010). The specific proteins that would be concentrated in such domains would depend on the type of membrane targeting sequence (Brown, 2006). For example, transmembrane proteins with a longer transmembrane domain that closely matches the increased thickness of the ordered phase bilayer would show affinity for these domains, as this would minimize the hydrophobic mismatch energy. Similarly, proteins that are post-translationally modified with long, saturated acyl chains would show affinity for ordered domains in the same way as saturated bilayer lipids themselves show ordered phase affinity.

While the coexistence of micron-scale, resolvable ordered and disordered phase lipid domains was readily observed in model membranes using fluorescence microscopy and phase-partitioning membrane probes (Simons and Vaz, 2004), no such structures have been observed in cell membranes. Although biochemical techniques such as detergent extraction continued to be used (London and Brown, 2000; Shogomori and Brown, 2003), the lack of direct imaging caused the lipid raft hypothesis to become controversial (Munro, 2003; Glebov and Nichols, 2004; Hancock, 2006) and the definition of a lipid raft has evolved over the years. Originally, lipid rafts were defined as “preferential packing of sphingolipids and cholesterol in moving platforms, or rafts, onto which specific proteins attach within the bilayer” (Simons and Ikonen, 1997). The lack of direct visualization resulted in an emphasis on the sub-diffraction-limited size of the domains such that they were described as being a “molecular complex in the membrane [that] consists of at least 3 molecules that includes a molecule with a saturated alkyl chain or a cholesterol molecule that plays a critical role in the formation of the complex itself” (Kusumi et al., 2004). An example of a yet later definition emphasizes the dynamic nature of the domains defining rafts as “small (10–200 nm), heterogeneous, highly dynamic, sterol and sphingolipids-enriched domains that compartmentalize cellular processes. Small rafts can sometimes be stabilized to form larger platforms through protein–protein or protein–lipid interactions” (Pike, 2006). The frequent modifications of the lipid raft hypothesis have questioned its validity but the hypothesis was undoubtedly the snowball that triggered new thinking and the emergence of new membrane models. Its emphasis on lipids was the motivation to develop new tools for lipid research. However, it should be kept in mind that organelle and plasma membranes of cells contain an extremely high protein density (Takamori et al., 2006; Dupuy and Engelman, 2008). Therefore one should not simply envisage cell membranes as systems where proteins floating in a “sea” of lipids. Instead, the membrane must be treated as a “lipid–protein composite” in which a very high density of transmembrane domains may impose order on nearby lipids complimenting lipid domains organizing proteins (Jacobson et al., 2007).

In some of these definitions, a substantial cohesion length that is a characteristic of a phase in model membranes is no longer included so that no distinction between nano-scaled complexes and meso-scaled domains are made. This lack of distinction may make it difficult to translate findings from pure lipid bilayers to complex cell membranes because the lack of microscopically visible lipid domains in cells is not proof of the absence of lipid rafts. Whether complexes of a few molecules could indeed be called a phase is biophysically controversial and for this reason, we continue to distinguish between multi-molecular complexes and meso-scale domains. Although this limit is arbitrary, meso-scaled domains should be above 20 nm in size and thus contain several thousand lipids (Pralle et al., 2000).

Defining lipid phases is not an issue in model membranes and thus lipid phase have been precisely mapped in such systems resulting in phase diagrams that show the phase behavior at different lipid compositions (Bezlyepkina et al., 2013). It is now recognized that the composition of the plasma membrane of cells in most cell types lies close to the critical composition for the liquid-ordered, liquid-disordered phase transition of lipid mixtures containing pure unsaturated phosphatidylcholine, sphingomyelin, and cholesterol (Lingwood et al., 2008). This may be a mechanism by which small changes in composition or environmental factors can cause large changes in organization. This was recently observed when resolvable sterol-enriched domains were found to form in the vacuole membrane of yeast cells in response to physiological changes, such as pH (Toulmay and Prinz, 2013).

Despite the lack of direct observation of lipid phases in intact and live cells, ordered-phase membrane domains are thought to play a role in a wide range of cellular processes, mainly in signaling at the plasma membrane and the selective trafficking of lipid components. We have used polarity sensitive membrane dyes, such as Laurdan, to quantify membrane order *ex vivo* and *in vivo* in intact zebrafish embryos (Owen et al., 2010a, 2012b). Even though fluidity differences in the plasma membrane are readily observed between cell types and cellular conditions, clear evidence of lipid phases in cell membrane could not be obtained with diffraction-limited imaging (Gaus et al., 2003). However, correlations between membrane order and cell functions were established. For example during T cell activation, high membrane order has been shown to be required for the correct localization of membrane-associated proteins and efficient T cell signaling (Rentero et al., 2008; Ventimiglia and Alonso, 2013). Membranes of high order were localized at the periphery of T cell synapse which is associated with actin and adhesion proteins, indicating a link between lipid organization and the actin cytoskeleton (Owen et al., 2010b). In addition, sub-synaptic vesicles with a high membrane order have also been observed, which may be important in the trafficking of specific T cell components, such as the raft-associated adaptor protein linker for activation of T cells (LAT; Williamson et al., 2011). Lipid rafts have similarly been implicated in various aspects of B cell signaling (Gupta and DeFranco, 2007). Other roles for highly ordered membrane domains include focal adhesions (Gaus et al., 2006) and cell migration (Gomez-Mouton et al., 2004), virus entry and budding (Mañes et al., 2000; Carrasco et al., 2004; Khurana et al., 2007; Lorizate et al., 2009), autoimmune disease (Jury et al., 2007; Miguel et al., 2011), the blood-brain barrier (Dodelet-Devillers et al., 2009), hormone signaling (Márquez et al., 2006; Yang et al., 2010) and in the trafficking of lipids in polarized cells (van Meer et al., 1987).

Most of the work to define lipid rafts experimentally has been conducted in artificial membranes, mammalian cells (both primary and cell lines) and yeast. Progress has also been made in analyzing membrane domains in plant cells. This has included the observation that detergent resistant membranes extracted from plant cell membranes (Peskan et al., 2000) which were found to be enriched in sterols and sphingolipids, similar to mammalian cells (Borner et al., 2005). This finding was later the subject of several reviews (Martin et al., 2005; Grennan, 2007). The similarity of plasma membrane order properties between plant and mammalian cells was reinforced by the observation that the membrane fluidity of bacteria, plant, mammalian and fungal membrane properties may display convergent evolution to a similar level regardless of membrane composition between species (Kaiser et al., 2011).

## NEW INSIGHTS FROM SUPER-RESOLUTION IMAGING

Much of the controversy surrounding lipid rafts developed as a result of their supposed small size which made them impossible to image using standard fluorescence microscopy approaches. This is because the resolution of a conventional fluorescence microscope is limited by diffraction to above 200 nm. However, in recent years, three families of techniques have emerged which all break the diffraction barrier and allow imaging of cellular structures far below the conventional 200 nm limit. These methodologies are structured illumination microscopy (SIM), stimulated emission depletion microscopy (STED) and photoactivated localization microscopy (PALM). Many of these techniques and now starting to be applied to imaging plant cells (Fitzgibbon et al., 2010; Kleine-Vehn et al., 2011). The major advantages and disadvantages of the techniques discussed here are summed up in **Table 1** for typical biological samples.

**Table 1:** Summary of super-resolution imaging techniques to probe membrane organization below the diffraction limit.

	PALM/STORM	STED	SIM	NSOM
Lateral resolution	20–30 nm	60–100 nm	100–120 nm	20–30 nm
Image speed	Minutes	Seconds	Seconds	Seconds
Image and sample geometry	2D or 3D image of fluorophores close to coverslip	2D image at any focal plane	3D image over entire cell	Only surface proteins
Equipment complexity	Simple	Complex	Intermediate	Complex
Analysis complexity	Complex	Simple	Intermediate	Intermediate

In SIM, the sample is illuminated with a grid pattern, which is then shifted while multiple images are acquired. A super-resolution image is then calculated computationally from the data. SIM can achieve resolutions of around 100 nm in lateral direction, can perform 3D imaging in live cells (although this is still technically challenging) and uses conventional fluorophores (Gustafsson, 2000; Kner et al., 2009; Shao et al., 2011).

Stimulated emission depletion microscopy uses a doughnut-shaped depletion laser beam to de-excite fluorophores at the periphery of a confocal excitation spot. This narrows the size of the spot thereby increasing the resolution. Depending on what laser powers the sample can tolerate from the depletion beam, resolutions of 50–100 nm laterally are typically possible in biological samples. The technique is built on a conventional laser-scanning microscope and has been applied to live cell imaging (Hell and Wichmann, 1994; Hein et al., 2008; Vicidomini et al., 2011).

Photoactivated localization microscopy and related techniques image and localize individual fluorophores, which typically results in localization precisions of individual molecules of around 20–30 nm. While the technique has long acquisition times and is generally a 2D technique based on total internal reflection fluorescence (TIRF) illumination, progress is being made in establishing 3D PALM as well as higher-speed imaging for live cell analysis (Betzig et al., 2006; Rust et al., 2006; Klein et al., 2011).

**FIGURE 1:**
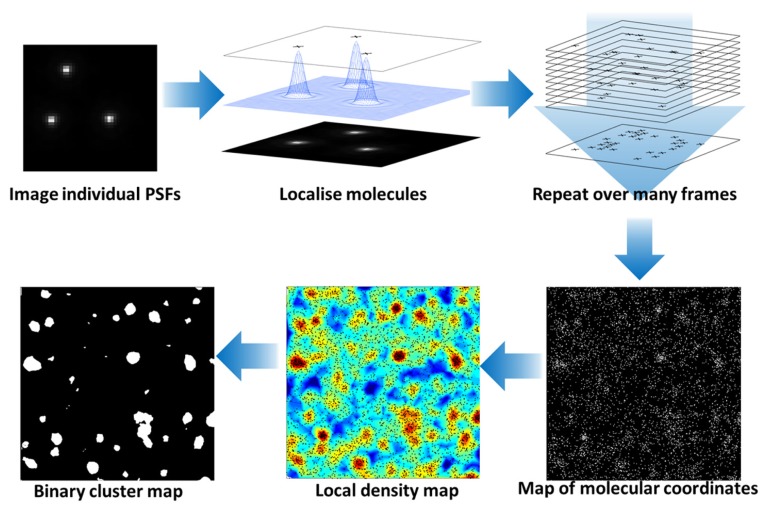
**Photoactivated localization microscopy analysis of protein clustering at the cell surface.** Photo-activation or stable dark states of fluorophores are exploited to limit the number of fluorescent molecules in each image frame. The fluorescence of individual molecules are captured with a camera and the center of the point-spread function (PSF) calculated to localize the molecules with nanometer precision. During the imaging processes, the fluorescence molecules are bleached so that the combination of photo-activation and photo-bleaching gives the appearance that molecules “blink” during the acquistion. Over successive frames, an image of all fluorescent molecular positions is built up. The molecular coordinates can be used to generate an image and be quantitatively analyzed to reveal the local density of fluorescent molecules (here based on Ripley K-function before and after application of a threshold) and hence clusters of proteins at the plasma membrane identified. For details on the cluster analysis, please see Williamson et al. (2011).

These methods have delivered previously unattainable data on membrane lipid domains and any proteins have been shown to be clustered within the plane of the membrane using super-resolution methods which otherwise appear homogeneous in conventional resolution systems (Owen et al., 2012a). PALM (**Figure 1**) can be used to map the localization of raft and non-raft targeted fluorescent fusion proteins and a quantitative analysis can distinguish protein clusters from random distributions, frequently identifying clusters on scale of 50–100 nm (Owen et al., 2010c, 2012a,c; Sengupta et al., 2011; Sengupta and Lippincott-Schwartz, 2012). One of the earliest single-molecule super-resolution data demonstrated the nano-scale clustering of Hemagglutinin (Hess et al., 2007), which is thought to cluster in lipid rafts (Takeda et al., 2003) and was more recently shown to cluster in an actin-dependent manner (Gudheti et al., 2013). Sengupta et al. (2011) used PALM and pair-correlation analysis to show that glycosylphosphatidylinositol (GPI)-anchored proteins formed nano-clusters there were sensitive to the cellular levels of cholesterol and sphingomyelin and cross-correlated with actin after antibody cross-linking (Sengupta et al., 2011). Similarly, we used a distribution analysis based on Ripley K-function to quantify the non-random distribution of membrane proteins (Owen et al., 2010c) and identified for example that the conformational states of the kinase Lck can regulate clustering, thereby linking intramolecular arrangement to intermolecular patterning (Rossy et al., 2013). However, it is not clear to which extent protein clustering reflects the underlying lipid organization. In unpublished data, we found that even weak protein–protein interactions induced by the fluorescent protein mEOS2 could cause clustering of raft-favoring and non-raft lipid anchors independently of the membrane fluidity. This suggests that protein interactions could easily override that partitioning preference of a protein into lipid phases. Hence localizing proteins may not be sufficient to map the distribution and geometry of lipid domains in cell membranes. To our knowledge, there are currently no lipid probes available that could be used to map lipid domains in cell membranes with PALM. Since the partitioning of fluorescent lipids into liquid-order and liquid-disordered phases differs markedly whether phases in model membranes or cell-derived membrane vesicles are examined (Sezgin et al., 2012), one can also not solely rely on the distribution of different lipids to map lipid domains. Hence more sophisticated lipid probes are needed to utilize the localization power of PALM to image lipid domains.

**FIGURE 2:**
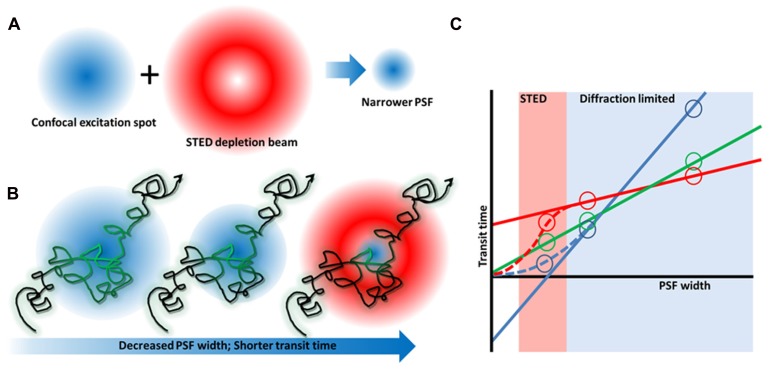
**Stimulated emission depletion microscopy microscopy and FCS diffusion laws.** **(A)** By combining a red-shifted, “doughnut”-shaped depletion beam with a confocal laser beam, the excitation spot and hence PSF can be narrowed. **(B)** FCS measures the time fluorescent molecules take to diffuse through the focus of a stationary beam. By decreasing the PSF width, the transit time becomes shorter so that even short lived complexes can be detected whose existence is canceled out when a larger observation area is used (Wawrezinieck et al., 2005). **(C)** By varying the PSF width, a plot of transit time vs spot size can be generated that reveals free diffusion (green), or membrane heterogeneity caused by an actin meshwork (blue), or membrane domains (red).

Excitingly, super-resolution microscopy also has the ability to generate new information on molecular dynamics. STED has been combined with fluorescence correlation spectroscopy (FCS) – a method for determining molecular diffusion coefficients based on fluorescence fluctuation analysis (**Figure 2**). This allows dynamics to be analyzed on sub-resolution length scales similar to what has been achieved previously with near-field scanning optical microscopy (NSOM) based techniques (Vobornik et al., 2008), but with a controllable spot size. Using STED FCS in cells, it was shown that sphingolipids and glycophosphatidylinositols (two putative raft markers) become transiently arrested in the plasma membrane whereas phosphoglycerolipids (non-raft molecules) do not. This trapping was cholesterol dependent, occurred in ~20 nm areas and lasted on the order of tens of milliseconds (Eggeling et al., 2009). A similar observation using STED FCS was also shown for cytoskeletal-dependent transient trapping (Mueller et al., 2011). Interestingly, a modified saturated phosphoethanolamine could be used to map liquid ordered domains in model systems below the diffraction limit (Honigmann et al., 2013) but showed no trapping in cells (C. Eggeling, personal communication). Collectively, the STED FCS data in cell membranes point more toward lipid complexes that are short lived, rather than lipid domains that may be positionally and temporally stable. Chemical modification of lipids may affect their dynamics and complex formation and hence like PALM, this super-resolution technique also depends on the availability of well-characterized probes for lipid research.

We would like to point out that near field scanning-type imaging approaches such as NSOM can analyze membrane organization at smaller length scales than are possible using conventional microscopy. In NSOM, the effects of diffraction are circumvented by placing the detector (typically a fiber) very close (much less that the wavelength of light) to the sample, detecting the emitted fluorescence and then raster-scanning to build up an image which can result in lateral resolutions of less than 10 nm. For example, this technique has been used to show that GPI-anchored proteins, commonly used as lipid raft markers, are arranged in nano-scale clusters on the surface of immune cells (van Zanten et al., 2009). These “hotspots” were found to be essential for integrin-based cell adhesion. In T cells, NSOM was used to detect clusters of CD3, CD4, and CD8 membrane proteins on the cell surface on nano- and meso- length-scales (Zhong et al., 2009). In a similar study, NSOM showed that the nanoscale organization of proteins and lipids in T cells was temperature dependent (Chen et al., 2009), consistent with the classical lipid raft hypothesis and the observation of cold-induced activation of T cells (Magee et al., 2005). Similar to STED, NSOM has also been paired with FCS to reveal differences in anomalous diffusion of phosphoethanolamine and sphingomyelin (Manzo et al., 2011).

Although not a super-resolution technique, we recently used fluorescence lifetime imaging microscopy (FLIM) to gain insights into lipid organization in cell membranes below the diffraction limit. This was possible because we used an unbiased unmixing approach, the so-called phasor apporach, to map the spectral signatures of Laurdan in each pixel. We could show that Laurdan in the plasma membrane of HeLa show is not a homogenous phase of intermediate order but a mixture of ordered and disordered domains. By using the pure lipid mixtures of 70:30 sphingomyelin:cholesterol and 100% dioleoylphosphatidylcholine as reference points for liquid-ordered and liquid-disordered phases, we estimated that ~76% of the plasma membrane is covered with ordered phases. This approach could not tell us whether Laurdan with an ordered FLIM signature comes from a continuous phase or from many domains and complexes with a large variation in sizes, simply because the data acquisition was still diffraction limited. One should also take into consideration that the liquid-ordered and liquid-disordered membranes in cells may have significantly different properties than the pure lipid mixtures that we used as reference data. It was for example shown that the difference in membrane order between phase-separated ordered and disordered domains in plasma membrane vesicles was much smaller than the differences observed in model membranes (Kaiser et al., 2009). However, combining environmentally sensitive probes with super-resolution technique may allow us for the first time to directly measure the bilayer properties of cell membranes. Unfortunately with Laurdan, this is not possible since it neither has a stable dark state for PALM nor is it STED-compatible due its fast photo-bleaching. But with more environmentally sensitive probes being developed (Bacia et al., 2004), we remain hopeful to one day characterize and map lipid complexes and domains in cell membranes.

## THE EFFECT OF THE ACTIN CYTOSKELETON

One of the biggest changes to our current understanding of membrane heterogeneity has been an elevation of the role of the cytoskeleton (Edidin, 2006). The cortical actin mesh has frequently been a target for new super-resolution based imaging methods, for example 3D PALM (Xu et al., 2012, 2013), SIM (Brown et al., 2011) and STED (Rak et al., 2011). The density and dynamics of the cortical actin network make this structure a defining feature of cell membranes.

**FIGURE 3:**
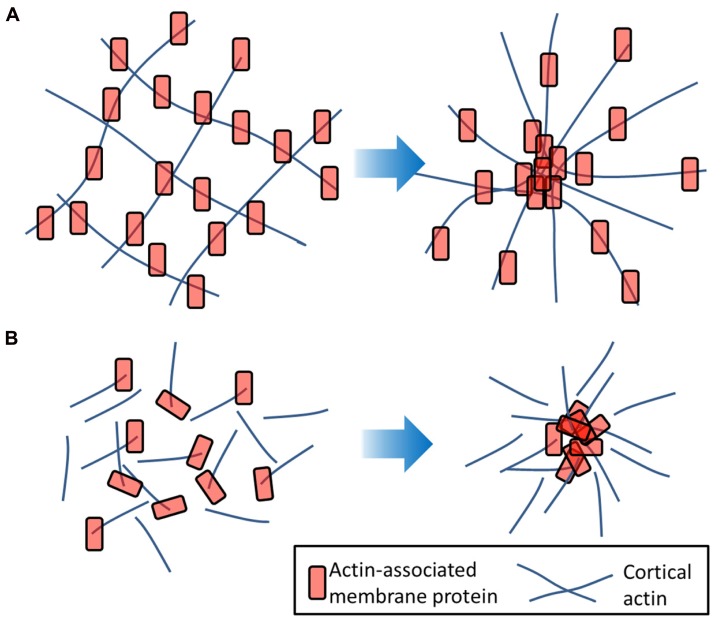
**Actin-induced clustering of membrane proteins.** **(A)** Membrane molecules (red) which are tethered to the underlying actin filaments (blue) may undergo transient clustering as the actin mesh flexes. **(B)** Proteins anchored to short, dynamic actin strands may undergo clustering in response to actin aster formation that requires a motor such as myosin and is therefore an energy-dependent process.

Firstly, the cytoskeleton can directly influence the diffusion and clustering of membrane proteins. The main theory here is the “picket fence” or “hop diffusion” model first developed by Kusumi et al. (2005). This theory holds that the cortical actin cytoskeleton forms a meshwork under the plasma membrane to which it is anchored by actin and bilayer-associated proteins. Molecules diffusing in the plasma membrane encounter these proteins as barriers causing them to be trapped in so called “transient confinement zones.” From time-to-time, lipids and proteins may be able to “hop” over these barriers thereby becoming trapped in a new zone (Fujiwara et al., 2002; Kusumi et al., 2005; Morone et al., 2006). Such compartmentalization would be a size-dependent process where proteins containing a large intracellular domain or transmembrane proteins would experience a greater barrier to diffusion caused by the underlying mesh (Heinemann et al., 2013). Where membrane proteins are linked to the dynamic cortical actin mesh, it has been shown that fluctuations in the cytoskeleton can cause transient focusing (clustering) of the plasma membrane proteins (Chaudhuri et al., 2011) as the actin grid spacing fluctuates. Actin-tethered membrane proteins may also form clusters *via* short, dynamic actin fibers aligning assembling into aster formations (Gowrishankar et al., 2012; **Figure 3**).

It has recently been shown that many membrane proteins have their diffusion and distributions regulated by cortical actin (Gudheti et al., 2013; Mattila et al., 2013). While much of the early work on hop diffusion was performed using extremely high speed single molecule and single particle tracking to map confinement zones, this area has also proves fertile for the use of variable spot-size FCS (**Figure 2**). By performing FCS experiments over a range of size scales, it is possible to infer information on the underlying, sub-resolution organization without requiring more complex super-resolution hardware. These so called “FCS diffusion laws” make it possible to determine whether it is transient confinement zones or membrane lipid domains that exert the greatest influence on diffusion within the bilayer (Lenne et al., 2006; Lasserre et al., 2008). For example, the lipid ganglioside GM1, one of the archetypal lipid raft components is influenced mainly by lipid domains, whereas the large transmembrane protein Transferrin-1 has strong interactions with the cytoskeletal meshwork (Wawrezinieck et al., 2005).

It may also be the case that the cytoskeleton causes an increase in membrane lipid order (the abundance of the liquid-ordered phase) and therefore influences diffusion and distributions indirectly by regulating the bilayer phase behavior. Blocking actin polymerization using latrunculin causes a decrease in membrane order observed with the environmentally sensitive membrane probe di-4-ANEPPDHQ (Jin et al., 2006). Membrane order was also low in plasma membrane blebs in which the bilayer had been detached from the underlying cytoskeleton. Stabilization of the actin meshwork using jasplakinolide had the opposite effect and caused an increase in membrane order (Dinic et al., 2013).

**FIGURE 4:**
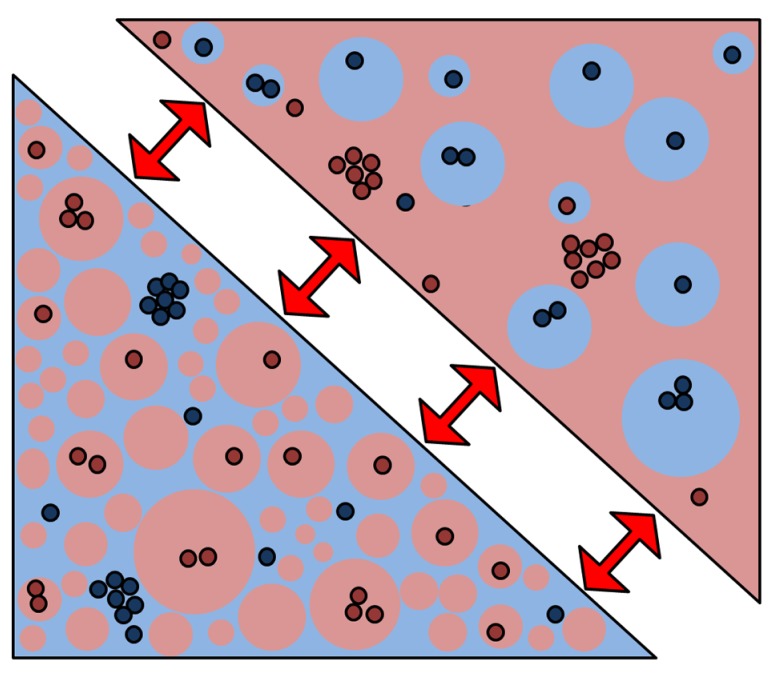
**A new membrane model based on percolating phase switching.** A new membrane model emerged from our data that indicates that the plasma membrane is indeed a mixture of ordered (red) and disordered (blue) phases where the ordered phase is the majority. Without changing the fraction, protein interactions may be regulated when the geometry of the phases switches (red arrows) from the percolating to the “island” phase. In so-called critical fluctuations, even small perturbations can trigger large-scale changes such as phase geometry.

It has been hypothesized that the cytoskeleton may cause “pinning” of local membranes in an ordered state, which then act as nucleation sites for the development of ordered-phase domains. Using computer modeling, it was demonstrated that if such pinning took place in a membrane that was very close to the critical composition for fluid – fluid phase coexistence, small critical fluctuations could cause many of the properties attributed to rafts, such as their small size and transient nature (Machta et al., 2011). Moreover, these critical fluctuations caused the formation of transient channels within the plans of the membrane, which could potentially regulate the interactions of membrane proteins over multiple length scales. This fits with the recent observation that the plasma membrane of cells contains a much higher coverage of the ordered phase than previously thought (Owen et al., 2012c) so that interactions may be controlled by which phase is the percolating “sea” phase and which phase represents the “islands” (**Figure 4**). While we have do direct evidence that phase geometry frequently change in cell membranes, coverage of 30–70% of either phase afford the possibility that protein interactions occur during the meso-scaled remodeling of phase geometries.

The high level of ordered-phase coverage could be the result of the extremely high density of membrane proteins (estimated at 23% protein coverage for the red blood cell membrane; Dupuy and Engelman, 2008) in the bilayer which impose order on the surrounding 1–2 shells of lipids adjacent to the protein (Jacobson et al., 2007). Such is the typical density of transmembrane domains (Takamori et al., 2006) that the membrane can be considered a lipid-protein composite and therefore the lipid properties may be dominated by transmembrane proteins (Jacobson et al., 2007). The switching of the “percolating” to the “island” phase could allow large changes in organization in response to very small changes in the physical environment (Lingwood et al., 2008). In this model, the partitioning of proteins into distinct phases is no longer the controller of specific interactions that then take place under static conditions. Instead, the switching of the percolating phase would allow selective mixing of components and hence would provide dynamic regulation. Such changing connectivity of different membrane domains and sub-regions has previously been observed by NSOM microscopy and in silico simulations (van Zanten et al., 2010). If such percolating phase switching indeed takes place, the dynamic properties of the cortical actin undoubtedly play a role.

## CONCLUSION

Newly developed imaging techniques which allow super-resolution are dramatically increasing our understanding of the complexity of cell membrane organization. While the basic principles of the original lipid raft hypothesis – ordered membranes based on cholesterol and saturated lipids – may remain, more details have already emerged that cause the distinction between lipid domains into which certain proteins may partition and lipid complexes that may contain multiple proteins. Other forces at work include direct protein–protein interactions, ordering of shell lipids by protein transmembrane domains, critical transient lipid composition fluctuations and a complex interplay between the bilayer and the underlying actin cytoskeletal meshwork. This structure may influence the distribution of membrane proteins directly or *via* its effects on membrane lipid order. Further technological advances, particularly the development of functional probes that report on the membrane environment are undoubtedly needed to answer many of the outstanding questions of the organizational hierarchy of cellular membranes. What started as one hypothesis that brought lipids back into the focus has now evolved into a number of competing membrane models that are not mutually exclusive. Excitingly, as we understand more of how cell membranes are organized, we also gain deeper insight into functional processes such as receptor signaling and cargo-driven endocytosis.

## Conflict of Interest Statement

The authors declare that the research was conducted in the absence of any commercial or financial relationships that could be construed as a potential conflict of interest.
